# Mass Spectrometry Strategies for *O*-Glycoproteomics

**DOI:** 10.3390/cells13050394

**Published:** 2024-02-25

**Authors:** Amanda Helms, Jennifer S. Brodbelt

**Affiliations:** Department of Chemistry, The University of Texas at Austin, Austin, TX 78712, USA; amanda.helms@utexas.edu

**Keywords:** glycoproteomics, tandem mass spectrometry, glycoprotease, enrichment, glycans, analytical workflow

## Abstract

Glycoproteomics has accelerated in recent decades owing to numerous innovations in the analytical workflow. In particular, new mass spectrometry strategies have contributed to inroads in *O*-glycoproteomics, a field that lags behind *N*-glycoproteomics due to several unique challenges associated with the complexity of *O*-glycosylation. This review will focus on progress in sample preparation, enrichment strategies, and MS/MS techniques for the identification and characterization of *O*-glycoproteins.

## 1. Introduction

**Scheme 1:**
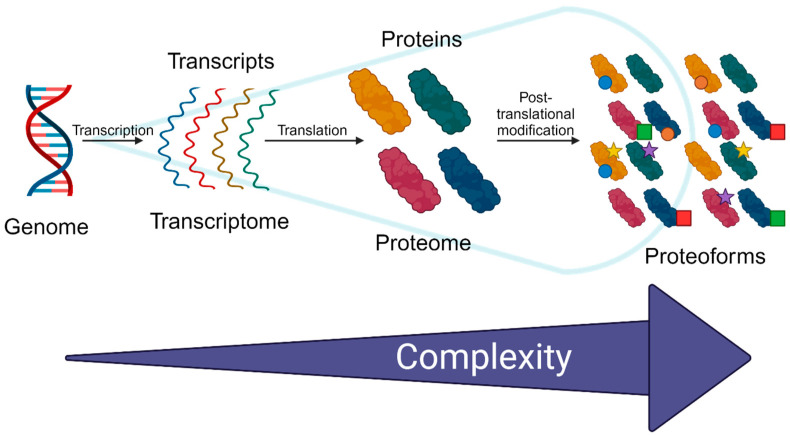
The increasing complexing of the gene transcription/translation process, particularly with the addition of post-translational modifications (PTMs) (represented by the various symbols attached to the proteins on the right). The dynamic interplay of PTMs increases the possible number of proteoforms.

Mass spectrometry stands as a premier tool for the identification and characterization of biomolecules, encompassing proteins [1,2,3,4], lipids [5,6,7,8,9], metabolites [10,11,12,13], glycans [14,15,16,17,18,19,20,21,22], DNA [23,24,25,26], and other small molecules [27]. In particular, new technologies and methodologies in mass spectrometry have had a tremendous impact on the field of proteomics, providing new insight into protein functions, interactions, localization, and reactivities. Advances in high performance mass spectrometers, [28] enrichment and separation strategies [29], ion activation methods [30], and data processing tools [31] have been instrumental in uncovering pivotal post-translational modifications (PTMs) that profoundly influence the structure and functions of proteins [2,32,33,34,35,36,37], and, in turn, the signaling of these proteins and interactions with other proteins. PTMs span a diverse array of functional groups and types of moieties, ranging from relatively small modifications like methylation and acetylation to the attachment of entire proteins (e.g., ubiquitination) [38,39,40,41,42,43,44]. Furthermore, modifications are dynamic and can be appended or removed throughout a protein’s life cycle. The addition or removal of PTMs regulate protein folding [35,45], mediate stability [46,47], modulate biological activity [48,49,50], and earmark proteins for degradation [37,49,51], among other functions, culminating in over a million proteoforms so far identified for the human genome (Scheme 1).

Deciphering how the locations and types of PTMs correlate with changes in protein structure and function poses an enormous challenge. Notably, glycosylation, one of the most prevalent and diverse types of protein modifications, can influence inflammatory responses, contribute to metastasis of cancer cells, and consequently serve as biomarkers for many conditions and diseases [14,15,16,17,21]. Additionally, disease progression may be tracked by abnormal modifications, notably glycosylation and/or heightened occurrences of specific glycan moieties, such as sialylations [18,50,52,53,54]. Owing to the rapid advances in the fields of glycobiology and glycomics and the substantial impact on fundamental biological processes and ultimately human health, this overview will focus on the key role of mass spectrometry in the characterization of glycoproteins, particularly emphasizing *O*-glycoproteomics. While the development of new mass spectrometry strategies has advanced both the fields of *N-* and *O*-glycoproteomics, *O*-glycoproteomics remains the greater challenge owing to the diverse complexity of *O-*glycans. Thus, this review will highlight key aspects of *O*-glycoproteomics, in some cases drawing from advances and applications in *N-*glycoproteomics that illustrate inroads that are not yet achieved for *O*-glycoproteomics.

## 2. Glycosylation

As a complex co- and post-translational modification, glycosylation plays numerous roles within and on the surface of cells [55,56,57,58,59]. Glycans are responsible for mediating cell-cell interactions [60,61], protein folding [62,63], and activating or inhibiting downstream processes within cells [64,65,66]. Additionally, glycans are common disease biomarkers but can be difficult to deduce owing to the complexity of combinatorial glycosylation and its role in disease progression [67,68,69]. Composed of mono- or oligosaccharides, the latter which are frequently multi-branched, glycans exhibit great microheterogeneity that increases proteome complexity as a single glycosite can host many different glycans [70,71]. Furthermore, multiple glycosites can exist in proximity or throughout the protein, resulting in numerous combinations that confound analysis. In addition, there are multiple types of glycosylation, including asparagine-linked glycosylation, known as *N*-glycosylation, and serine- or threonine-linked glycosylation, namely *O*-glycosylation [72,73], as well as *C*-glycosylation [74], in which an anomeric bond is made between two carbon atoms, typically used to develop *O*-glycoside mimics, and *S*-glycosylation [75]. Linked to the sulfur atoms on cysteine residues, *S*-glycosylation is less common than *N*- and *O*-glycosylation and is found in bioorganisms like bacteriocins [76].

### 2.1. N-Glycosylation

**Scheme 2:**
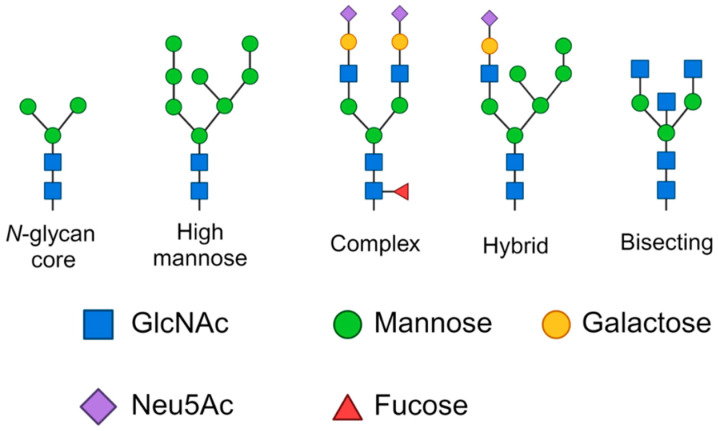
Depiction of the *N*-glycan core and each of the four *N*-glycan subclasses.

*N*-linked glycans—glycans attached to asparagine (Asn, N) residues in a predictable motif of N-X-S/T, where X is any amino acid other than proline—are a highly studied class of PTM as they are extremely diverse and prevalent in mammalian proteomics [72]. *N*-glycans have a conserved core structure, allowing them to be easily targeted by enzymes that cleave and thus release the *N*-glycans from Asn side-chains of glycoproteins. The released glycans can be analyzed after separation from the proteins (albeit losing the site-specific context on the proteins) [72,77] or glycoproteins can be digested to product *N*-glycopeptides (as well as other non-glycosylated peptides). The conserved core includes two *N*-acetylglucosamine (GlcNAc) and three mannose (Man) sugars (Scheme 2). From this core structure, four main types of more elaborate glycans can be synthesized: high mannose, complex, hybrid, and bisecting.

### 2.2. O-Glycosylation

*O*-glycosylation presents significant technical hurdles whether studied as released glycans or still attached to proteins [73,78]. One contributing complexity to *O*-glycosylation is the different array of originating saccharides that are linked to serine and threonine residues. *N*-acetylglucosamine glycosylation (*O*-GlcNAcylation) serves important roles in cellular processes, akin to its other *O*-glycosylation counterparts, most strikingly serving as nutrient response, which contributes to other significant changes within the cellular environment [79]. In addition, *O*-GlcNAcylation was discovered to exist outside of traditional glycosylation pathways and is controlled by the actions of two enzymes, which add or remove the monosaccharide from proteins [80]. Unlike *O*-GlcNAcylation, the linkage of mannose to serine and threonine does take place in the endoplasmic reticulum followed by further modifications in the Golgi apparatus, akin to traditional glycosylation. This type of glycosylation is most heavily linked to α-dystroglycan, which when disrupted has been linked to muscular dystrophy [81]. Finally, mucin type *O*-glycosylation refers to glycosylation that originates with a *N*-acetylgalactosamine (GalNAc) bound to serine or threonine side chains and will be the primary focus of this review.

**Scheme 3:**
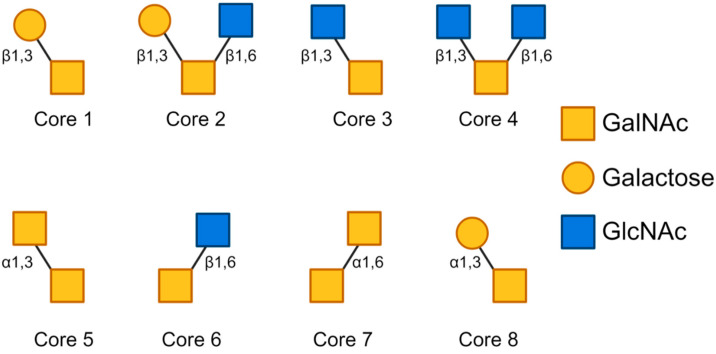
*O*-glycans have eight canonical core structures. Some only differ by the linkage between two saccharides.

**Scheme 4:**
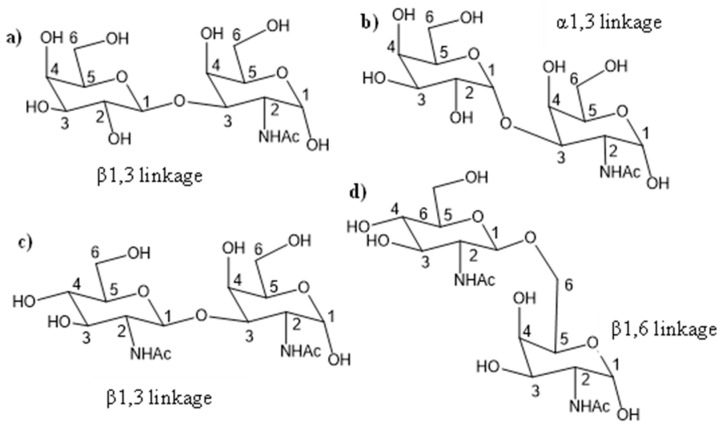
Representative linkages between a handful of core saccharides are shown. Alpha linkages refer to linkages between an axial carbon on the anomeric saccharide (the non-reducing end) and a carbon on the adjacent saccharide. Beta linkages are those in which anomeric carbon is on the same face as the sixth position carbon. The numbers correspond to the carbon positions that are involved in the glycosidic bond. Typically, the first number is the carbon position of the saccharide closer to the non-reducing end of the glycan (or colloquially to the left in representative renditions) and the second number is the carbon position of the saccharide closer to the reducing end of the saccharide (or to the right in drawings).

Mucin *O*-glycans are known to exhibit eight confirmed core structures, with the initial four (core 1 to core 4) being the most prevalent (Scheme 3) [82]. Some of the core structures differ only by the linkage position (Scheme 4) but have profound structural and functional effects on the proteins they modify, as do linkages at the non-reducing end of the glycan [83,84,85]. For example, certain linkages, like an α2,6 linkage connecting sialic acid and GalNAc, are more closely associated with cancer metastasis, whereas an α2,3 linkage between sialic acid and galactose is more commonly affiliated with leukocyte trafficking [86]. Development of enzymes capable of cleaving each individual type of *O*-glycan core or cleaving all of them collectively from proteins has not been accomplished. Furthermore, unlike their *N*-linked counterparts, predicting *O*-glycosites is challenging owing to the absence of a defined protein sequence binding motif [73]. *O*-glycosylation may occur at any Ser or Thr residue. While some prediction software tools have aimed to address this puzzle of site localization, the experimental results can differ from prediction [87]. Furthermore, multiple glycosites can exist in proximity or even adjacent to one another, introducing macroheterogeneity that is unseen in *N*-glycosylation. Moreover, considering the potential scope of glycan microheterogeneity at each site, disentangling the patterns, and interpreting the outcomes remains an obstacle despite ongoing efforts to alleviate this burden [88,89,90]. Notwithstanding these challenges, creating comprehensive inventories of glycosylation sites and the associated *O*-glycans is imperative as they significantly modulate cellular processes [16,65,73,78,83,85,91].

Often *N*- and *O*-glycosylation work in tandem within the cell but participate in different roles; for example, *O*-glycosylation is responsible for receptor function during cell-cell interactions, depending on glycosite and structure [78]. Harmonious glycosylation is particularly vital in the context of immune responses, as aberrant *N*- or *O*-glycosylation is implicated in the pathogenesis of chronic inflammation and various cancers [92]. Glycoproteins can exhibit both types of glycosylation (*O*- and *N*-) simultaneously and dynamically, further complicating the assignment of the resulting combinatorial patterns of modifications and the ability to link them to functional or structural roles. This interplay is especially evident in the immune response and viral fusion, in which diverse glycans facilitate cell-to-cell recognition in the glycan envelope and modulate protein folding to promote or inhibit activation site occupancy [59,66,93,94]. In addition, *O*-glycosylation influences the stability, solubility, and pharmacokinetic parameters of biotherapeutics [67,95,96,97]. This underscores the importance of the analysis of *O*-glycoproteomics: the ability to fully characterize glycoproteins is a key step towards understanding the effects of different glycans.

## 3. MS Workflows for Protein Analysis

Advances in analytical methods to facilitate *O*-glycoproteomics have sprung from innovative developments in mass spectrometry strategies, sample preparation methods, and data processing techniques. There have been a number of recent reviews summarizing some of these developments [78,88,98,99,100,101,102,103]. Here, we cover the principal workflows and three key facets of the workflow (MS/MS, sample preparation, and enrichment) that enable successful outcomes for glycoproteomics.

**Scheme 5:**
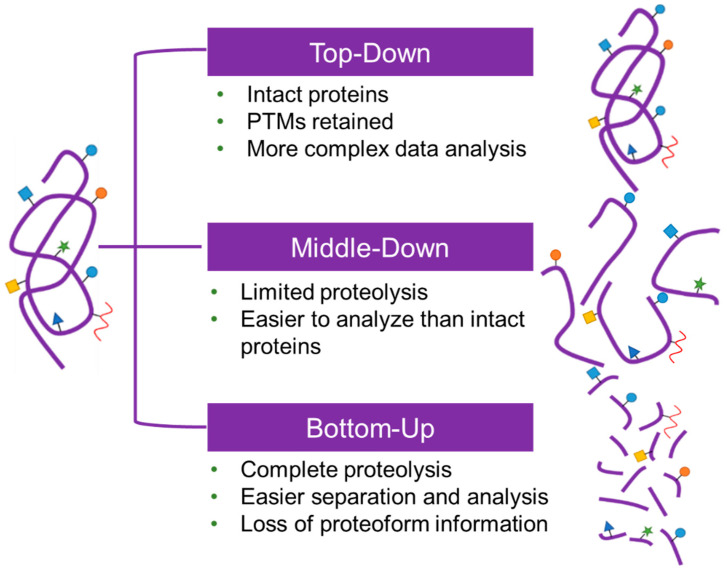
Comparison of workflows for protein analysis by mass spectrometry. Intact proteins are analyzed in top-down methods. Middle-down strategies use limited proteolysis or highly selective proteases to generate large peptides or sub-units of proteins. Bottom-up methods focus on analysis of peptides produced by proteolysis of proteins. The shapes attached to the protein or peptide segments are arbitrary representations of different PTMs.

Although there are many variations in experimental strategies for the mass spectrometry analysis of glycoproteins (i.e., variations in sample preparation, separation methods, MS/MS options, and data analysis), there are two primary categories of workflows: bottom-up and top-down (Scheme 5). Bottom-up methods utilize proteases to cleave proteins into peptides; top-down methods entail characterization of intact proteins. A third option, middle-down, represents a less commonly deployed intermediate between bottom-up and top-down methods. Each option offers advantages and disadvantages and may contribute new insights into the comprehensive characterization of glycoproteins.

### 3.1. Bottom-Up MS

As peptides are generally easier to separate, ionize, and dissociate than proteins, bottom-up methods are the most common ones used for all types of proteomic applications. Peptides may be generated by the digestion of proteins using a number of different proteases (i.e., trypsin, GluC, *O*-glycoproteases, etc.), and MS/MS spectra obtained for the peptides are used to identify the peptides, typically using sophisticated search algorithms to match the experimentally generated fragmentation patterns to ones generated in silico. The resulting peptides are matched to protein sequences, ultimately allowing the identification of proteins [104,105,106]. In the context of glycoproteomics, bottom-up methods are heavily favored over top-down approaches, especially for *O*-glycoproteomics because the micro- and macro-heterogeneity of *O*-glycosylation creates extreme complexity for the characterization of intact proteins [80,101,102,107,108,109].

**Scheme 6:**
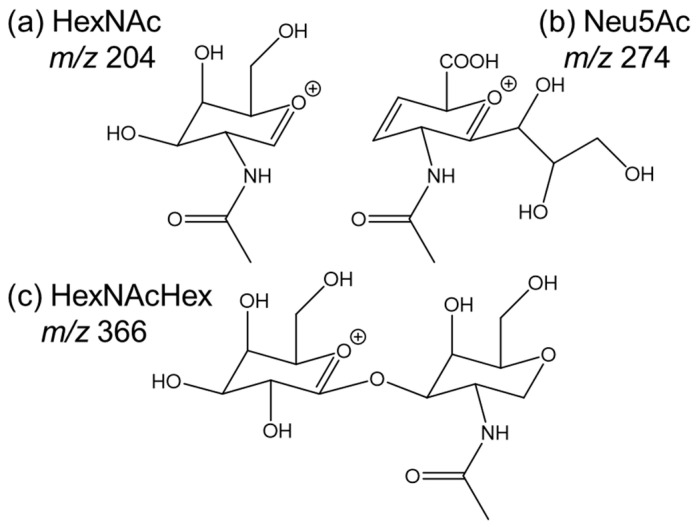
Examples of oxonium ions from mucin-type *O*-glycans, (**a**) HexNAc oxonium ion of *m*/*z* 204, (**b**) Neu5Ac oxonium ion of *m*/*z* 274, and (**c**) HexNAcHex oxonium ion of *m*/*z* 366.

**Figure 1:**
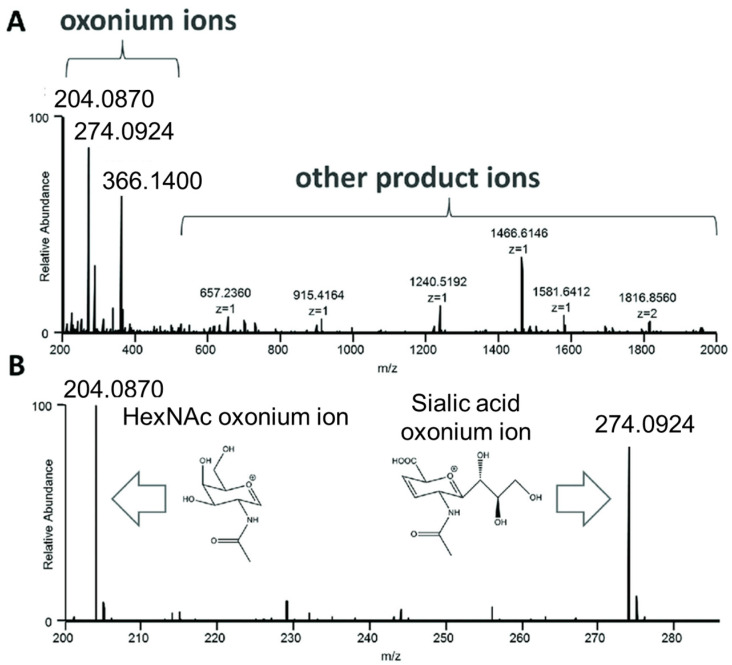
Oxonium ions are produced upon collisional activation of glycopeptides (implemented in a data-independent, all ion fragmentation mode) during LC-MS/MS analysis of a biotherapeutic protein. (**A**) Representative mass spectrum and (**B**) expansion of the lower *m*/*z* region populated by oxonium ions. Structures of the HexNAc and sialic acid oxonium ions are shown. Reprinted and adapted from Ref. [110], courtesy of Taylor and Francis Ltd. (www.tandfonline.com (accessed on 16 January 2024)).

As described in more detail later, an MS/MS analysis of *O*-glycopeptides may yield partial (incomplete), or in some cases more extensive, information about the peptide sequences, the modification sites, and the glycan compositions and structures. Given that multiple different glycans can be conjugated to each possible *O*-glycosite, the bottom-up workflow is helpful for identifying the glycan compositions that occupy a given glycosite, yielding a list of *O*-glycoforms. For a known protein sequence that is cleaved into peptides, the number of HexNAc, Hex, Neu5Ac, Neu5Gc, or Fuc saccharides appended to a specific peptide is readily determined based on the known masses of each of the saccharides and how they sum together to yield the resulting mass shift of the peptide. MS/MS analysis can be used to identify the overall composition of glycans via the detection of specific oxonium ions (Scheme 6) that correlate with known saccharide masses (i.e., *m*/*z* 366 for HexNAcHex ion). For example, tryptic *O*-glycopeptides from biotherapeutic proteins subjected to collisional activation generate several types of oxonium ions in the lower *m*/*z* range as displayed in the mass spectrum in Figure 1 [110]. In this example, the appearance of oxonium ions was used to screen for the presence or absence of the glycosylation of the subunits of different antibodies, monitor site-specific glycosylation, and quantify variations in multi-glycosylated biotherapeutics [110].

While insights about potential glycan compositions and structures may be derived from key principles of *O*-glycan biosynthesis [73,93,111], only general “theoretical” possibilities can be inferred without more detailed MS and MS/MS analysis, as discussed later. A comprehensive list of glycoforms is helpful for understanding glycosite occupancy and any trends with glycan structures for specific sites, such as the presence of sialylations or a preference for a specific core type. However, a confident characterization of glycoproteins based on glycopeptide profiles can be impeded by the presence of multiple glycosites in proximity (inhibiting proteolytic digestion or confounding site localization), the difficulty of analyzing longer peptides or ones with more complicated branched glycans, and the complexity of the suite of glycans present [78,80,108,112].

Much effort has been expended to increase the quality of data generated from bottom-up *O*-glycoproteomics workflows, including refining or combining separation methods (typically various modes of liquid chromatography (LC)) [14,22,68,98] and integrating ion mobility (IM), a type of gas-phase electrophoretic separation [68,113,114,115]. Although reversed-phase chromatography is by far the most commonly utilized mode for separation of peptides, other methods like hydrophilic interaction liquid chromatography (HILIC) [14,69,116,117], porous graphitized carbon [22,68,118,119,120], and strong anion exchange (SAX) [19,88,116,117,121] chromatography have been successfully implemented for the analysis of both *O*-glycopeptides and *O*-glycoproteins alike. Ion mobility adds another dimension of separation based on how an ion’s charge and shape influences its migration through a collision cell or drift tube in the gas phase [122,123]. Incorporating ion mobility in the workflow reduces the potential overlap of co-eluting glycopeptides and decreases the probability of generating chimera spectra from co-isolation and the activation of isomeric or isobaric peptides [68,113,114,115]. For example, in one recent study ion mobility was utilized to facilitate the distinction of various levels of isomerization in GalNAc glycans and confirm multiple glycosites for a single glycoform, as demonstrated for five isomers of MUC5AC-GalNAc mucin motif glycopeptides [115].

Bottom-up strategies that focus on an analysis of *O*-glycopeptides provide an overall view of the complex microheterogeneity of different glycosites and glycan compositions through the production and analysis of small, easy to characterize glycopeptides, reducing spectral complexity. However, this strive for simplification comes at the expense of losing contextual information about the potential combinatorial pattern of glycosylation that is derived from an examination of intact proteins. Both qualitative and quantitative combinatorial patterns of modifications are lost; this information is crucial for fully understanding the interplay of glycosylation and its code for protein function. Owing to this shortcoming, the allure of examining intact proteins has spurred the development of top-down methods to move closer to achieving global glycoproteomics.

### 3.2. Top-Down MS

Top-down mass spectrometry entails the analysis of intact proteins [42,124,125,126]. Focusing on intact proteins offers the potential for the elucidation of the combinatorial patterns of PTMs, an opportunity not possible with bottom-up methods that digest proteins into peptides and thus lose the contextual ensemble of modifications. Obtaining a complete characterization of proteins requires the preservation of PTMs throughout the sample processing, enrichment, separation, and ionization steps, in addition to requiring the development of fragmentation (MS/MS) methods that retain labile modifications and afford sufficient information to identify sequences and localize PTMs. The use of high-performance mass spectrometers is essential to facilitate the assignment of fragment ions from intact proteins with high mass accuracy.

Advances in search algorithms, ion activation methods, and sample processing techniques have expanded the adoption of top-down MS with significant inroads achieved for the characterization of selected proteins in targeted studies [127,128] and some applications demonstrating high throughput global proteomics [125,126,129,130]. The challenges of attaining sufficient chromatographic resolution to separate isomeric or isobaric proteoforms and rapidly obtain comprehensive fragmentation patterns to assign sequences and localize PTMs with confidence remain formidable [129].

**Figure 2:**
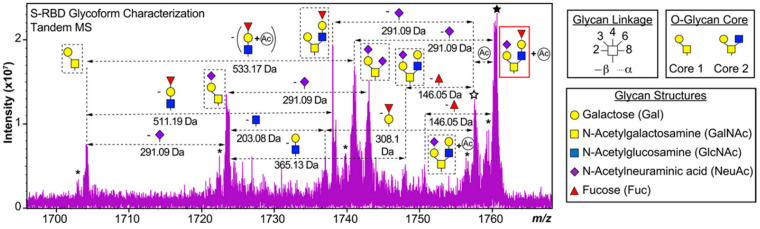
MS/MS characterization of SRBD proteoform (15+) isolated from the quadrupole window centered at *m*/*z* 1760.5. Assignments of the glycan structures are marked in the spectrum and illustrated in the legend shown on the right side. Glycoform characterization reveals the specific S-RBD proteoform to have a core 2 type GalNAcGal(GalNeuAc)(GlcNAcGalFuc) glycan. Neutral loss glycan products are labeled. N-Terminal acetylation (Ac) is labeled and corresponds to a +42 Da mass shift. The black star represents the 15+ charge state precursor ion corresponding to *m*/*z* 1760.5, and the white star represents the 15+ charge state precursor ion corresponding to *m*/*z* 1757.7 (−Ac). The asterisk “*” denotes an oxonium ion loss. Reprinted and adapted from Ref. [131].

Top-down MS has not been widely used for glycoproteomics, largely owing to the heterogeneity of glycosylation, which complicates the separation of glycoforms and localization of glycosites. Moreover, the fragmentation patterns obtained for intact proteins reveal few details about the branching patterns of the glycans, even if the glycan compositions can be estimated based on high accuracy mass measurements. Only a handful of studies have reported the use of top-down methods for the analysis of *O*-glycoproteins [131,132,133,134]. For instance, rich fragmentation is observed as illustrated for one representative MS/MS spectrum of the regional-binding domain (RBD) of the spike (S) protein of SARS-CoV-2 in Figure 2 using a high performance FTICR mass spectrometer [131]. Collisionally activated dissociation (CAD) and electron capture dissociation (ECD) were used in combination to extend the depth of the characterization of the RBD. The MS/MS spectrum of the core 2 glycan revealed the structure of the glycan of the glycosylated RBD [131]. Eight *O*-glycoforms were characterized using this method [131].

The characterization of intact glycoforms through top-down MS/MS reveals the heterogeneity that complicates the analysis of *O*-glycoproteins. Top-down MS/MS was used to identify *O*-glycoforms from emerging viral variants, such as the Omicron variant of SARS-CoV-2, and the results were compared to wild type and Delta variants of the protein, showing the evolution of viruses through increased glycosylation across the receptor binding domain [132]. Interestingly, some of these findings differ from those reported in another study that examined the glycosylation of Alpha, Beta, Delta, Gamma, and wild-type variants of the RBD using a combination of conventional bottom-up glycoproteomics and glycomics methods [135]. Additional comparative studies of top-down and bottom-up methods are warranted to identify the facets of the experimental workflows (sample preparation and introduction, enrichment, search strategies) that might account for variations in outcomes, particularly when profiling the distributions and patterns of post-translational modifications like glycosylation. Other top-down studies of *O*-glycoproteins have aimed to map the glycome for the identification of trends in glycosylation of fetuin from three sources (human serum, bovine serum, and recombinant human fetuin) [133] and to characterize the glycoprotein heterogeneity of biotherapeutic proteins [134]. Additionally, MALDI with in-source decay implemented on a high performance mass spectrometer platform has also been used for the analysis of intact glycoproteins, either for the glycotyping of biological samples or determination of the oligosaccharide repeat units of bacterial glycoconjugate vaccines [136,137].

There remains substantial room for growth in the field of top-down glycoprotein analysis, particularly to address the microheterogeneity of glycans that makes their structural dissection so arduous. However, advances in ion activation methods and data analysis methods offer many avenues for improving the outcomes of top-down approaches.

### 3.3. Middle-Down MS

Akin to bottom-up proteomics, middle-down MS is accomplished through the aid of proteases to digest proteins into more easily characterized peptides. The middle-down strategy entails limited proteolysis, aiming to produce peptides larger than ~3 kDa, followed by MS/MS to characterize the large peptides or subunits [138]. The analysis of larger peptides offers a technically easier alternative to top-down methods and may preserve some information about combinatorial patterns of PTMs owing to the greater lengths of the peptides. Although the middle-down approach offers a compelling intermediate to bottom-up and top-down methods, it has been rarely used in the context of glycoproteomics. In one study, native MS and middle-down methods were combined to investigate the structure of plasma glycoproteins, particularly focusing on the *N-*glycosylation sites of human erythropoietin and properdin [139]. Middle-down strategies have been more commonly used for the characterization of biotherapeutics, such as monoclonal antibodies, by cleaving the antibodies into smaller Fc and Fab subunits for a streamlined analysis of *N*-glycoforms [140,141,142]. While still relatively unexplored, middle-down MS has the potential to identify combinatorial glycosylation on large peptides and may yield insight into new data analysis strategies that could be elevated for top-down methods.

### 3.4. Tandem Mass Spectrometry for Glycoprotein Analysis

One of the most important advances required for expanding the breadth and depth of information derived from the mass spectrometry analysis of glycoproteins is the development of improved or alternative ion activation/dissociation techniques. Creating reproducible and interpretable patterns of fragment ions holds the key to the assignment of peptide or protein sequences and localization and identification of modifications. Many ion activation/dissociation techniques have been applied in the field of glycoproteomics as part of bottom-up or top-down strategies, as described in the following sections.

### 3.5. Collision Induced Dissociation

**Scheme 7:**
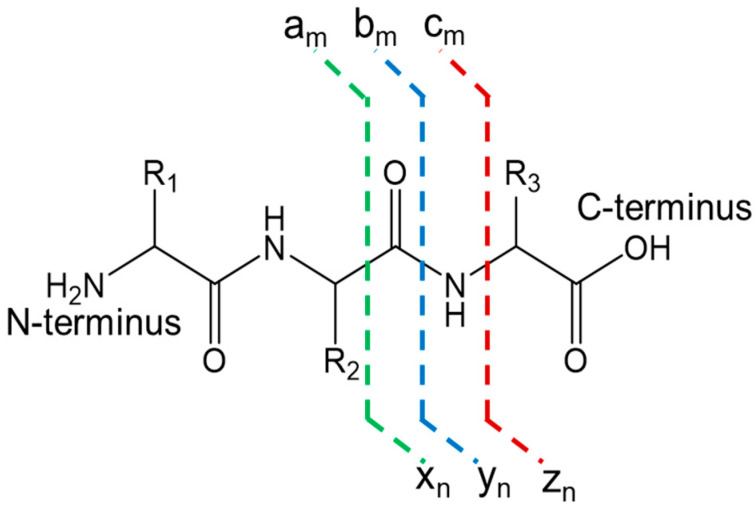
Array of backbone cleavages and resulting fragment ion types produced by different ion activation methods. *a*/*x* ions originate from cleavage of the C_a_-C bonds (UVPD only) of the peptide or protein backbone; *b*/*y* ions are produced from cleavage of the amide bond (HCD, EThcD, UVPD), and *c*/*z* ions are released upon cleavage of the N-C_a_ bonds (ETD/ECD, EThcD, UVPD). The subscript number represents the number of amino acids contained in the fragment ion.

Collision induced dissociation (CID) is a longstanding powerhouse of tandem MS and by far the most popular ion activation method on all commercial mass spectrometers. CID primarily cleaves the amide bond between amino acids, the most labile bonds in the peptide backbone, generating *b-* and *y-*type ions that provide a fingerprint for the identification of sequences (Scheme 7) [30,143]. CID can be performed in a number of ways—for example, the collision process can be implemented in quadrupole devices that serve as a collision cell or in various ion traps; it can entail a few or many collisions, and the collisions may be classified as low or high energy depending on the kinetic energy of the precursor ions. These variations alter the energy deposition and efficiency of CID but ultimately achieve the same goal of adding energy to cause fragmentation. CID is the most widely used ion activation method for bottom-up proteomics workflows [144] and has also been applied for the analysis of small proteins, such as histones, in top-down studies [145]. The performance of CID diminishes with the molecular size of the peptide or protein owing to the broad re-distribution of internal energy over many vibrational modes, thus explaining why CID has shown more limited success for large proteins. Moreover, the covalent bonds that anchor PTMs to the side-chains of amino acids are often labile and thus are frequently broken during collisional activation, releasing the modifications [98]. The prevalent cleavage of PTMs impedes their localization on peptides or proteins. However, the modulation of the collision energy used for CID offers topology information about the *O*-linked glycans, even yielding some characterization of the glycans in some cases, as well as peptide sequence ions [146]. This strategy of incrementally increasing CID energy to generate fragments that originate from the cleavage of glycosidic bonds and peptide backbone cleavages has been applied for the characterization of several *O*-glycoproteins, including bovine fetuin and bovine kappa-casein [146].

**Scheme 8:**
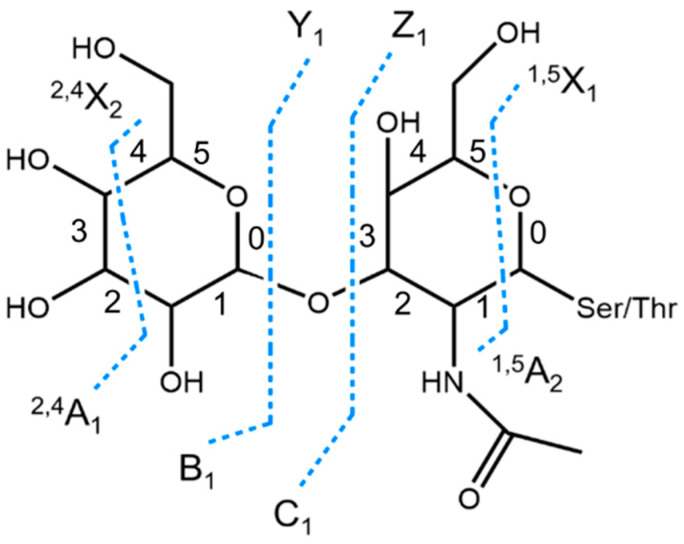
Glycan cleavages and resulting ion types. The bonds of the sugars are numbered starting at the bond between the oxygen on the six-membered ring and carbon one (bond 0) and then continuing clockwise around the ring, resulting in six bond cleavages total. The super-script numbers for the cross-ring cleavage ions designate the two sugar bonds that are broken.

Some glycoproteomics strategies deliberately process the glycoproteins using endoglycosidases, such as PNGase F, to release the glycans, allowing their collection and separate analysis by MS/MS methods. This general approach is known as glycomics [147,148] and is primarily aimed at deciphering the compositions and patterns of the glycans. Although glycomics does not retain site-specific information to facilitate the localization of the glycans on the proteins, it provides detailed insight into the glycan profiles. The CID of glycans predominantly results in production of B and Y ions which originate from cleavage of the labile glycosidic bond between saccharide units (Scheme 8). Y ions contain the oxygen of the glycosidic bond and the reducing end of the glycan, whereas the B ions contain the non-reducing end of the glycan. Both fragments are informative as they help reconstruct the composition of the glycans. Additionally, the reverse fragments, C and Z ions, where C retains the glycosidic oxygen and Z does not, are also generated by higher energy CID methods [84].

Cross-ring cleavages, entailing cleavages between carbon-carbon bonds or the carbon-oxygen bond on the six-membered ring, allow the localization of intersaccharide linkages, which disambiguate glycan isomers from one another [84]. Cross-ring cleavages result in A/X-type ions (Scheme 8) and are generally rare in the CID spectra of glycopeptides; however, CID has been shown recently to produce cross-ring cleavages of released high mannose *N*-glycans [149].

The CID of glycopeptides or glycans also generates oxonium ions (as shown earlier in Scheme 6 and Figure 1); these are intact or truncated mono- or disaccharide ions that are helpful in the identification of the broad sugar types present in the glycans [98,150,151]. Oxonium ions are indicative of the saccharides present and are often used for glycopeptide targeted analysis [152], a strategy in which the detection of oxonium ions upon CID is used to pre-screen glycopeptides and trigger a second stage of ion activation for a more thorough characterization of the glycopeptides, as demonstrated recently for the analysis of human IgG and *E. coli* glycoproteins [152], crustacean neuropeptides [153], mouse tissues [154], and *O*-glycoprotein mixtures [155].

Other refinements of CID methods have also afforded a more detailed characterization of *O*-glycopeptides. One notable improvement is the application of stepped-energy CID, in which peptides are activated using multiple separate collision energies [156]. All of the resulting fragment ions are combined in a CID spectrum, yielding more extensive information as some fragments are only produced at certain energies [156]. Stepped CID techniques have also been employed for *O*-glycan analysis, such as to determine the glycan structures of viral proteins [157], deduce glycosylation patterns of viral proteins [158], and to profile the diversity of glycan expression on bovine mucin glycoproteins [159]. Additionally, stepped CID was used to uncover new *O*- and *N*- glycosites of fibronectin and fill in overlooked sialylation information, offering a step forward in supporting functional studies of fibronectin [160]. Another recent study probed the glycosylation landscape of SARS-CoV-2 based on the rich fragmentation patterns of the glycopeptides generated from a stepped HCD strategy [161].

While CID is virtually unsurpassed for sequencing peptides, it is not well suited for the characterization of large proteins owing to a lack of sufficient fragmentation throughout the sequence. In this regard, there have been less than a handful of studies that have used CID for the characterization of intact *N*- and *O*-glycoproteins via top-down strategies [131,162,163,164], and, as briefly described in the earlier section, on top-down MS analysis [127,128,132,133,134].

### 3.6. Electron-Based Dissociation

In the context of peptide-based bottom-up methods for glycoproteomics, CID has been widely employed, but the prevalent cleavage of the labile glycans has hampered their localization, an outcome that has motivated the development of other ion activation methods that enhance the preservation of the glycans, such as electron-based techniques. The energy deposition that causes the activation/fragmentation of ions in electron-based techniques is derived from the exothermicity of an electron transfer from a reagent ion (either an electron or an electron-donating ion) to a positively charged analyte (i.e., peptide or protein). This class of activation method includes electron transfer dissociation (ETD) [165] and electron capture dissociation (ECD) [166], and a pivotal feature of these methods that is distinctive from CID is the far more efficient retention of PTMs during the analysis of peptides or proteins. Although the specific mechanisms are different, both ETD and ECD produce *c-* and *z-*ions via the cleavage of N-C_α_ backbone bonds (Scheme 7).

The performance of electron-based methods is generally enhanced by the application of supplemental activation [167,168,169,170,171], which increases the conversion of non-dissociated precursor ions into fragment ions as well as facilitating the separation of fragment ions that otherwise remain paired with each other via non-covalent interactions. The net result is a substantial increase in fragmentation efficiency and the production of a more diverse array of fragment ions. One premier example is the hybrid technique known as electron transfer-higher energy collision dissociation (EThcD) [165], which combines ETD with supplemental collisional activation. In this approach, a selected precursor ion is subjected to ETD, and all of the resulting product ions, including both surviving and charge-reduced precursors, are subjected to CID which causes additional energization and fragmentation of all ions. These additional fragmentation pathways allow an enhanced characterization of proteins and localization of associated PTMs [101,172]. EThcD has been extensively applied to glycomics and glycoproteomics alike, generally affording a better characterization of glycopeptides in cases where CID or ETD alone has faltered [168,173,174,175,176,177,178].

**Figure 3:**
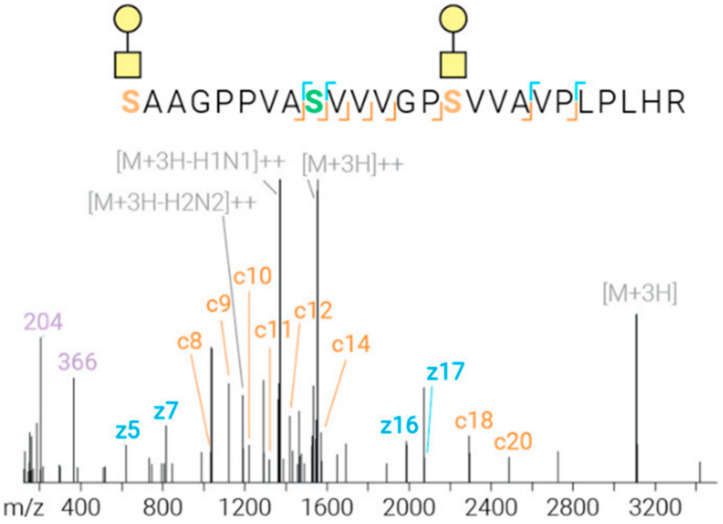
EThcD spectrum of a doubly glycosylated *O*-glycopeptide from a missed OpeRATOR cleavage of bovine fetuin that has both an N-terminal *O*-glycosite site and a second internal *O*-glycosite. Oxonium ions are labeled in purple, C-terminal *z*-type fragments in blue, N-terminal *c*-type fragments in orange, and whole glycopeptide ions or glycopeptide fragment ions with some saccharides missing are in gray. Reprinted and adapted from Ref. [155].

EThcD has proven particularly valuable in localizing glycosites, primarily owing to its ability to preserve the glycan moieties during fragmentation and thus produce peptide sequence ions that retain the glycans as exhibited by characteristic mass shifts. An example of an EThcD mass spectrum of a doubly-glycosylated *O*-glycopeptide generated upon the digestion of bovine fetuin using the *O*-glycoprotease OpeRATOR is shown in Figure 3 [155]. There are three possible *O*-glycosites (S1, S9, S15) on this 25 residue peptide. The mass shifts of the *c_18_* and *c_20_* fragment ions suggest the presence of two HexNAcHex glycan moieties on the peptide. The *c_9_* fragment ion reveals that the second glycan is not located at S9 because the net mass shift of *c_9_* remains the same as the mass shift of the *c_8_* ion, confirming one HexNAcHex glycan at the N-terminal S1. Additionally, the *z_16_* and *z_17_* fragment ions confirm that the second glycosylation site occurs on the C-terminal half of the peptide, restricting it to S15 [155]. The ability to localize multiple glycans on a single *O*-glycopeptide showcases the benefits of EThcD.

These benefits of EThcD were also vital for identifying novel *O*-glycosites and uncovering possible binding motifs on the spike protein of SARS-CoV-2 [158]. EThcD was also utilized for a site-specific analysis of unusually *O*-glycosylated Fc fusion proteins, in which glycopeptide linkers containing pentose instead of a reducing end GalNAc were identified [175]. The additional fragment ions generated by EThcD often allow a precise localization of the glycans to specific amino acid residues, such as when profiling amyloid precursor proteins to understand their role in Alzheimer’s pathogenesis [179], identifying novel *O*-glycosites in biotherapeutics [176], and for use in global *O*-glycoproteome workflows to differentiate glycosylation patterns between healthy and diseased patients [180]. The structural information obtained with EThcD bolsters its position as a powerful technique for glycoproteomics as EThcD has proven to be a premier method for localizing glycosites [174] and deciphering information about the branching patterns of the glycans [174]. Moreover, its applicability to high-throughput studies has positioned EThcD as a leading technique in the field of *O*-glycoproteomics, as demonstrated in one exemplary study in which multiple glycosites were identified on a single glycopeptide from human serum, allowing a quantitative analysis of the glycopeptides and deducing biomarkers for IgA nephropathy [178].

Despite its many attributes, EThcD does not consistently promote cross-ring cleavages. Cross-ring cleavages are vital for determining the positions of glycosidic bonds, enabling a complete structural characterization of glycan structures. This shortcoming of EThcD has promoted the development of other ion activation methods.

### 3.7. Ultraviolet Photodissociation

Ultraviolet photodissociation (UVPD) offers another alternative ion activation mode, generating an array of fragment ion types (*a*, *b*, *c*, *x*, *y*, and *z*) of peptides and proteins. The activation process of UVPD is faster than CID and deposits a larger amount of internal energy, elevating ions to excited electronic states and thus allowing access to unique fragmentation routes with higher energy barriers [36]. The dissociation of peptides or proteins directly from the excited electronic states results in *a*- and *x-*ions that are unique to UVPD (see Scheme 7) [36]. Owing to the fast high energy deposition, labile modifications such as glycans are not preferentially cleaved, allowing their localization via the detection of product ions that retain the modifications. In the context of glycoproteomics, UVPD promotes far more numerous cross-ring cleavages than other dissociation techniques [181,182,183,184,185,186]. Because the characterization of glycan structures is necessary for an unambiguous identification of exact glycoforms, UVPD offers a promising approach.

The abundant cross-ring cleavages (A and X ions, with A containing the non-reducing end and X the reducing end of the glycan) generated from UVPD allow a confident determination of the carbon positions of glycosidic bonds between saccharide units, a feature that is crucial for determining *O*-glycan core structures, some of which vary only by linkage position. Without this information, linkages between saccharide units are ambiguous, even if inferred based on prior knowledge about glycan synthesis in the endoplasmic reticulum or the Golgi apparatus [63]. This facet is also beneficial for determining linkages between non-reducing end saccharides. For example, glycan antenna can be asymmetrical or symmetrical depending on which saccharides are present, making it challenging to pinpoint those on each branch [151]. Additionally, linkages between the periphery saccharides are also difficult to discern. For instance, several types of sialic acid linkages have been identified, with the most prominent being the α2,3 and α2,6 linkages between sialic acid and galactose [187]. With UVPD, cross-ring cleavages are generated that can distinguish the two based upon the position of the cleavage [188].

**Figure 4:**
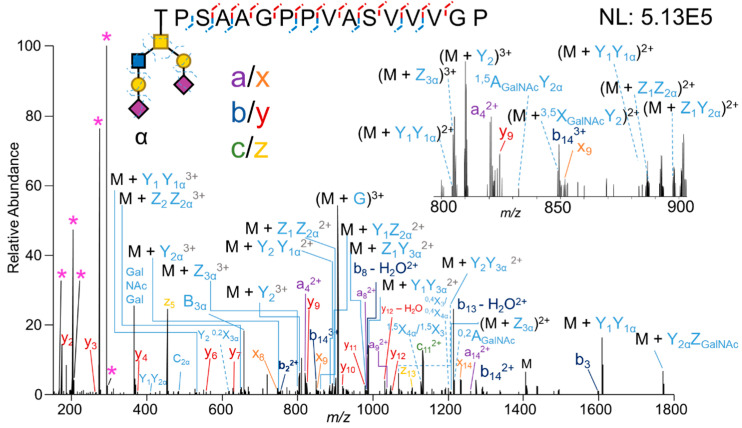
UVPD mass spectrum (acquired using 3 mJ per pulse, 8 laser pulses) of T(+1312.45 Da)PSAAGPPVASVVVGP (3+, *m*/z 906.75), an *O*-glycopeptide from bovine fetuin generated with glycoprotease IMPa. The oxonium ions in the low *m*/*z* region are labelled with pink asterisks. Glycan-specific fragment ions are labeled in light blue. Those pertaining to the peptide are in black. Ions labelled in bold font are bracketing ions that confirm glycan localization. Labels with dashed marker lines refer to fragment ions that incorporate cross-ring cleavages. M represents the entire peptide sequence and G represents the entire glycan. Sialic acids are shown on the glycan structure using purple diamonds [188].

One example showcasing the identification of glycan structures and characterization of the peptide sequence is showcased in Figure 4 for the analysis of the *O*-glycopeptide T(+1312.45)PSAAGPPVASVVVGP by UVPD [188]. This peptide was generated by proteolysis of bovine fetuin using glycoprotease IMPa. Not only is the glycan localized to the N-terminus based on detection of mass-shifted b_2_ and b_3_ ions, but there are several glycan-containing fragment ions, such as B_3α_ (*m*/*z* 657.23), Z_2_Z_3α_ (*m*/*z* 713.26), C_2_ (*m*/*z* 472.17), and (M + Y_2_Y_1α_)^2+^ (*m*/*z* 885.45), that confirm the branching pattern. Additionally, two cross-ring cleavage ions, ^0,3^X_2_Z_1α_ (*m*/*z* 567.2) and ^3,5^X_2_Y_4α_ (*m*/*z* 966.34), confirm the α2,6 linkage between the galactose and sialic acid on the shorter branch of the glycan, as these linkages do not retain the sialic acid, but contain an empty third carbon position. The extensive fragmentation pattern displayed in Figure 4 underscores the ability of UVPD to confirm the exact structure of *O*-glycans.

Despite the attributes of UVPD, it has not been as widely utilized as EThcD. While some commercial mass spectrometers are now equipped with UVPD, software tools for spectral interpretation and fragment ion assignment lag behind, thus necessitating tedious and time-consuming manual interpretation. However, new algorithms [22,80,89,189,190] are emerging to address this issue and should accelerate a broader adoption of UVPD for *O*-glycoproteomics.

### 3.8. IRMPD

Infrared multiphoton dissociation (IRMPD) energizes ions via the cumulative absorption of many low energy IR photons. As such, it is a low energy activation method that causes cleavage of the weakest bonds, akin to CID, and thus also causes the cleavage of labile modifications like glycans. IRMPD has been utilized for the study of simple glycopeptides but has not been widely explored [191,192,193].

**Scheme 9:**
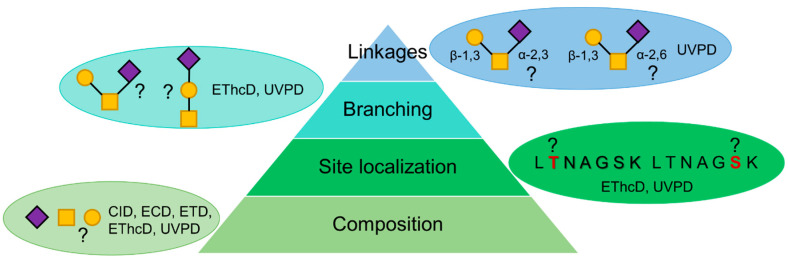
Levels of structure characterization achieved by collisional activation, electron-based activation, and UV photoactivation for a representative mucin type *O*-glycopeptide. GalNAc saccharides are represented by yellow squares, Gal as yellow circles, and Neu5Ac as purple diamonds, according to accepted glycan symbol nomenclature.

A comparison of the levels of information derived from each ion activation/dissociation method is shown in Scheme 9. Based upon the observation of the mass shifts of peptides that correspond to the attached saccharides and the production of oxonium ions, all dissociation techniques yield insight about the composition of a glycan on a glycopeptide. Glycosite localization requires preservation of the glycans on the peptide fragment ions, an outcome most feasible with electron activation methods and UVPD. The determination of branching patterns requires glycan cleavages, either detected as free partial glycan ions or partial glycans attached to peptides. The linkages between saccharide units are discerned by cross-ring cleavages.

## 4. Sample Preparation and Enrichment Methods

Glycosylation results in dynamic, heterogeneous populations of low abundance glycoproteins. To facilitate analysis by mass spectrometry there has been considerable effort to enhance the relative abundances of glycopeptides via selective enrichment methods and to enhance the ionization efficiencies via derivatization strategies. The most popular methods of sample preparation specific to *O*-glycoproteomics are described in the next sections.

### 4.1. Enzymatic Digestion of O-Glycoproteins

Numerous enzymes have been traditionally used to facilitate the success of mass spectrometry for bottom-up proteomics and glycomics [98]. For example, trypsin has been a landmark protease used to cut proteins after Arg and Lys residues, thus generating small peptides well-suited for mass spectrometry workflows [104]. Endoglycosidases, exoglycosidases, and glycoproteases have all played prominent roles in advancing the field of glycoproteomics. Exoglycosidases are enzymes that remove monosaccharides from the non-reducing ends of *N-* or *O*-glycans, typically sialic acids [67,71,84,98,194]; this is usually performed prior to digestion with other enzymes. Endoglycosidases release entire glycans from the proteins [98]. Unlike PNGase F, a readily available endoglycosidase that effectively cleaves all *N*-glycans from *N*-glycoproteins [195], there is no universal enzyme that can remove all *O*-glycans appended to the serine or threonine residues of *O*-glycoproteins. However, there are a few commercial endoglycosidases that are able to cleave truncated core 1 *O*-glycans, and to a lesser extent, core 3 *O*-glycans [67,68,78,98,111,155,196,197]. Owing to the lack of suitable enzymes, chemical reactions, such as reductive β-elimination, have been used to cleave all glycans from *O*-glycoproteins [198,199]. The chemical methods may cause peeling reactions which clip the terminal monosaccharides. Such strategies have been modified to better preserve the reducing ends of released *O*-glycan and afford intact glycans [200].

*O*-glycoproteases are a class of enzymes that cleave the protein backbone adjacent to *O*-glycosylated serines or threonines. For example, the first commercially available *O*-glycoprotease, OpeRATOR (OgpA), cleaves N-terminal to unsialylated core 1 *O*-glycans [155]. While groundbreaking at its onset, OgpA has proven less effective for densely glycosylated proteins and requires additional sample preparation steps, such as desialylation prior to its usage. The removal of sialylations reduces the likelihood of finding potential biomarkers, as highly sialylated glycans are prominent in many disease states. While useful for cleaving proteins containing core 1 glycans, OgpA is not a universal solution, motivating the development of alternative enzymes more broadly adapted for the cleavage of *O*-glycoproteins for bottom-up workflows. As core 1 and core 2 *O*-glycans are commonly found in biotherapeutics and are highly relevant for biological applications related to mucin domain proteins, both ones that are clinical and endogenous, their analysis is imperative.

Other *O*-glycoproteases have since been discovered, including StcE, which cleaves between proximal glycosites, prior to the second occupied site in an S/T*-X-S/T* motif [201,202] (where the asterisks indicate glycosylation sites). Appropriately dubbed a mucinase, StcE efficiently digests densely glycosylated mucin proteins containing regions where half of all residues are either serine or threonine and all are capable of being glycosylated [201,202]. *O*-glycoproteases with other specificities have been reported, such as BT4244 and CPaA, [202,203] but have not been as widely used as StcE [201,202,204,205]. The emergence of immunomodulating metalloprotease (IMPa), which cleaves N-terminal to core 1 and core 2 glycans, has offered another significant advance for bottom-up *O*-glycoproteomics [82,188,206]. For example, the brain *O*-glycoproteome of mice was investigated for the first time by implementing IMPa into a global workflow in which a large cohort of sialylated *O*-glycans was discovered [207]. However, the performance of IMPa is suppressed for regions containing adjacent glycosites, in which cleavage is inhibited owing to steric hindrance. Another mucinase has recently emerged, SmE, which does not exhibit low proteolytic activity for more densely *O*-glycosylated regions of proteins [208]. In fact, early results suggest that SmE cleaves efficiently even between adjacent glycosites, as demonstrated for T cell immunoglobulin and mucin-domain containing (TIM) proteins [208]. The robust performance of SmE makes it a promising candidate for the characterization of densely *O*-glycosylated proteins [88]. The exploration of new candidates for a universal *O*-glycoprotease is a worthwhile endeavor, as it can be one of the greatest aids for *O*-glycoproteomics, similar to what universal glycoproteases have done for *N*-glycoproteomics.

### 4.2. Enrichment Strategies

The incorporation of enrichment methods into the *O*-glycoproteomic workflow is both commonplace and extremely strategic owing to the need to enhance the capture of low abundance glycopeptides overshadowed by unmodified peptides. Enrichment can be performed offline or online depending on the objective and includes a vast array of methods ranging from solid phase extraction to liquid chromatography.

As there are several enrichment strategies already developed for *N*-glycoproteomics, they do not always easily translate to *O*-glycopeptide enrichment, either due to the *O*-glycans small size compared to *N*-glycans, or due to differences in their binding affinities [19]. Thus, enrichment strategies amenable to *O*-glycoproteomics have been developed independently or adapted from existing enrichment protocols. Lectin enrichment is a method that has been employed for glycopeptide enrichment for years now. Lectins are carbohydrate binding proteins [209] that possess selective affinities for certain glycan moieties and can be used for *N*- and *O*-glycoprotein or glycopeptide enrichment. Typically immobilized on solid supports, lectins attach to the glycan moiety of their specific bias; for *O*-glycopeptides, this is commonly *Vicia villosa* agglutinin [108]. In order to enrich an array of glycan types, multiple lectins can be combined in a single enrichment step to capture as many glycan types as possible, or in succession, and then released. This strategy enables focused applications, such as screening for diagnostic markers of colorectal cancer [210] or for global studies of cancer *O*-glycoproteomes [211]. Owing to the preferential glycan bias associated with lectins, this enrichment method is most effectively employed for targeted applications. Thus, other enrichment techniques are often used in tandem or orthogonally with lectin methods [212]. Enrichment via capture by hydrazide beads has been a popular chemical enrichment strategy owing to its high specificity [213].

Antibody-based methods offer another strategy that has been employed to enrich *O*-glycopeptides with high specificity. As an example, recombinant anti-Tn monoclonal antibodies attached to beads have been used to capture and enrich Tn-containing glycoproteins from Colo205 cells via immunoprecipitation [214]. Antibodies that exhibit selectivity for the capture of proteins carrying *O*-GalNAc [215] or *O*-GlcNAc [216] glycans have also been successfully developed. Even more specialized antibodies, such as *O*-Tyr specific antibodies, have been produced and demonstrated for targeted applications [217].

Recently, hydrophilic interaction liquid chromatography (HILIC) emerged as a compelling enrichment technique for *O*-glycopeptides [218]. Owing to the hydrophilic properties of the glycan moieties appended to *O*-glycopeptides, HILIC allows a selective retention of glycosylated species while non-glycosylated peptides rapidly migrate through the stationary phase [108]. Because *N*-glycopeptides also interact with the HILIC media, the removal of *N*-glycans prior to HILIC separation is imperative to reduce complexity in the analysis of *O*-glycopeptides [213,219]. HILIC separation can be performed offline or online in the MS workflow, making it an attractive option for high-throughput studies. Additionally, HILIC has been shown to be compatible with a range of *O*-glycopeptides, such sialylated glycopeptides or those that contain less common saccharides like *O*-xylosylations [218]. Overall, HILIC offers selectivity, versatility, and sensitivity for *O*-glycopeptides and has been utilized for cancer biomarker discovery [98,100], general enrichment [68,109,212,213,220,221,222,223], and the glycosite analysis of mucin domain proteins [88,220], antibodies [67,224], viral glycoproteins [222], and other glycoproteins [100,109,213,221]. As a follow-up to HILIC, electrostatic repulsion hydrophilic chromatography (ERLIC) is a variation of HILIC that utilizes an anion-exchange column under HILIC solvent conditions to induce the separation of acidic and basic *O*-glycopeptides [21,225]. One study reported an increase in *O*-glycopeptide identifications, particularly bis- and tri-sialylated glycopeptides, following ERLIC fractionation [225].

Akin to conventional reversed phase liquid chromatography, porous graphitized carbon (PGC) separates analytes based on hydrophobicity and has been used for the enrichment and/or separation of *O*-glycopeptides [16,22,118,226]. Used as a solid phase extraction technique as well as an online separation method, PGC allows the separation of *N*- and *O*-glycopeptides in a straightforward workflow [227].

### 4.3. Ion Mobility Methods

As noted earlier, ion mobility offers a promising strategy for separating isobaric or isomeric glycopeptides in the gas phase based on their shape, size, and charge state [114]. Multiple methods of ion mobility spectrometry, including high-field asymmetric waveform ion mobility spectrometry (FAIMS) and differential mobility spectrometry (DMS), have been applied to enhance the separation of glycopeptide isomers [115,228,229]. Peptides that may have multiple possible glycosylation sites are difficult to separate using even high-performance chromatographic methods, and ion mobility offers an orthogonal option of dispersion. For example, a mixture of three isomeric *O*-glycopeptides, ones containing the same glycan located at different positions, were successfully separated by combining liquid chromatography with DMS along with MS/MS characterization using ECD and CID [229]. FAIMS has been implemented for an enhanced separation of glycopeptides, as demonstrated for the differentiation of isomeric mucin-modified peptides [115] and analysis of mixtures of glycopeptides derived from tryptic digestion of proteins extracted from human serum [228].

## 5. Conclusions

The field of *O*-glycoproteomics remains a rich frontier for mass spectrometry, offering both unmet technical challenges and promising great dividends for cracking high impact problems in glycobiology. The understanding of *O*-glycoproteomics has increased tremendously within the last few decades owing to advancements in high resolution mass spectrometers, as well as new enrichment and separation methods originating from the exploration of new stationary phases, such as PGC and ones used for HILIC. Additionally, the development of *O*-glycoproteases that selectively cleave glycoproteins near *O*-glycosites, thus assisting the targeted analysis of the resulting *O*-glycopeptides, has proven to be a powerful addition to the mass spectrometry workflow. Moreover, the use of new ion activation methods and multi-step MS/MS strategies, along with more versatile data processing tools, has expanded the structural information harvested. Increasing the comprehensive characterization of the micro- and macroheterogeneity of *O*-glycoproteins remains a major goal and will continue to inspire new analytical strategies in the upcoming decade.

## Data Availability

No new data were created or analyzed in this study. Data sharing is not applicable to this article.
